# A nomogram for predicting postoperative overall survival of patients with lung squamous cell carcinoma: A SEER-based study

**DOI:** 10.3389/fsurg.2023.1143035

**Published:** 2023-04-06

**Authors:** Jin Rao, Yue Yu, Li Zhang, Xuefu Wang, Pei Wang, Zhinong Wang

**Affiliations:** ^1^ Department of Cardiothoracic Surgery, Changzheng Hospital, Naval Medical University, Shanghai, China; ^2^ Medical College, Guangxi University, Nanning, China; ^3^ School of Health Science and Engineering, University of Shanghai for Science and Technology, Shanghai, China

**Keywords:** nomogram, lung squamous cell carcinoma (LSCC), surgery, prognosis, surveillance, epidemiology, end results (SEER)

## Abstract

**Background:**

Lung squamous cell carcinoma (LSCC) is a common subtype of non-small cell lung cancer. Our study aimed to construct and validate a nomogram for predicting overall survival (OS) for postoperative LSCC patients.

**Methods:**

A total of 8,078 patients eligible for recruitment between 2010 and 2015 were selected from the Surveillance, Epidemiology, and End Results database. Study outcomes were 1-, 2- and 3-year OS. Analyses performed included univariate and multivariate Cox regression, receiver operating characteristic (ROC) curve construction, calibration plotting, decision curve analysis (DCA) and Kaplan–Meier survival plotting.

**Results:**

Seven variables were selected to establish our predictive nomogram. Areas under the ROC curves were 0.658, 0.651 and 0.647 for the training cohort and 0.673, 0.667 and 0.658 for the validation cohort at 1-, 2- and 3-year time-points, respectively. Calibration curves confirmed satisfactory consistencies between nomogram-predicted and observed survival probabilities, while DCA confirmed significant clinical usefulness of our model. For risk stratification, patients were divided into three risk groups with significant differences in OS on Kaplan–Meier analysis (*P *< 0.001).

**Conclusion:**

Here, we designed and validated a prognostic nomogram for OS in postoperative LSCC patients. Application of our model in the clinical setting may assist clinicians in evaluating patient prognosis and providing highly individualized therapy.

## Introduction

Lung cancer is the predominant cause of cancer-related mortality worldwide with an estimated 2 million new cases and 1.76 million deaths annually (1, 2). Lung squamous cell carcinoma (LSCC) is among the most studied histological subtypes of non-small cell lung cancer (NSCLC) (3, 4). Compared to lung adenocarcinoma (LUAD), LSCC is associated with distinct epidemiological features, lack of effective targeted treatment options and a poor clinical prognosis (5). As such, development of a prognostic predictive model for this patient population could significantly facilitate implementation of individualized treatment strategies (6). To date, prognostic research has primarily focused on NSCLC; studies concerning LSCC are scarce (7). Furthermore, the 8th Edition Tumor, Node, and Metastasis (TNM) staging system formulated by the American Joint Committee on Cancer (AJCC) is currently used for predicting NSCLC patient prognosis, although only three parameters, tumor, node, and metastasis, are involved (8).

As graphic prediction tools, nomograms are widely used to evaluate the clinical prognosis of patients suffering various malignancies (9). Nomograms incorporate a variety of clinically significant factors to effectively predict the probability of events such as mortality risk or overall survival (OS) relevant to individual patient profiles (10). Although use of nomograms often guides clinical decision-making, no Surveillance, Epidemiology, and End Results (SEER)-based nomograms useful for predicting OS among postoperative LSCC patients have been reported to date.

Here, we utilized patient data obtained from the SEER database to construct a nomogram for predicting OS of postoperative LSCC patients.

## Patients and methods

### Study design, data sources and ethics statement

Here, we conducted a retrospective analysis of data obtained from the SEER database, which contains information concerning patient demographics, primary tumor site, tumor morphology, stage at diagnosis, first course of treatment as well as mortality outcomes pooled from 18 National Cancer Institute registries since 1973. Data concerning approximately 35% of the American population are included in the SEER database (11). We obtained permission for dataset access (authorization code: 11874-Nov2018). Data were extracted using SEER*Stat software 8.3.8 (http://seer.cancer.gov/seerstat/). Primary cancer classification was performed according to the International Classification of Diseases for Oncology, 3rd Edition (ICD-O-3), which identifies cancer categories according to primary site, histology, behavioral code and grade. Informed consent was waived as all SEER data contained no personally identifiable patient information. This study was conducted in accordance with the Declaration of Helsinki and the Harmonized Tripartite Guideline for Good Clinical Practice (International Council for Harmonisation). Due to the public nature of the SEER database no institutional review board approval was sought.

### Cohort selection

Eligibility criteria for inclusion in our study were as follows: (I) age >18 years old; (II) patients diagnosed with primary malignancy with tumor site codes C34.0–C34.9 from 2010 to 2015; (III) histology codes 8051, 8052, 8070–8076, 8078, 8083, 8084, 8090, 8094, 8120 and 8123 confirmed on pathology; (IV) patients who underwent operative management; T1-4N0-1M0 patients were exclusively enrolled. Exclusion criteria were as follows: (I) lack of data considered essential such as race, tumor grade, TNM classification, laterality, marital status, insurance status, tumor size and survival outcomes; (II) patients who died within 1 month after surgery to exclude the possible influence of postoperative complications; (III) patients with nodal (i.e., N2 and N3) or distal (i.e., M1) metastases as they generally were not eligible for surgery. Tumor staging for SEER database patients was performed in accordance with criteria set forth by the AJCC (12).

### Study covariates and outcomes

Patient baseline demographic data included age at diagnosis, sex, race, marital status and insurance status. Histopathologic tumor characteristics including primary site, laterality, grade, T classification and N classification were extracted from the SEER database. Therapeutic strategies employed in patient management including types of surgery, radiotherapy and/or chemotherapy were also extracted. An optimum cutoff value for categorizing patients into groups by age in years (<67, 67–77 and >77) was determined using X-tile software. When patient race was known, patients were classified as black, white or other (i.e., American Indian/Alaska Native or Asian/Pacific Islander). Patients were grouped according to tumor size (T1, T2, T3 or T4) and N stage (N0 or N1) parameters.

In this study, OS was selected as the endpoint of interest and defined as the time from diagnosis to deaths from all-causes. Causes of patient mortality were coded according to death certificate data. The SEER database is updated annually to include data concerning follow-up and prognosis. Here, the latest patient data considered was released in December 2016. Thus, survival time for censored observations was measured in months from diagnosis until death or last follow-up (December 31, 2016).

### Nomogram construction and validation

First, multivariable Cox regression models for 1-, 2- and 3-year OS with optimal predictive performance were incorporated into nomograms. The “plot” and “nom” functions in the rms package for R software were used. Time-dependent receiver operating characteristic (ROC) curves as well as corresponding areas under the curve (AUC) at 1-, 2-, and 3-year timepoints were generated to assess predictive accuracy. Calibration curves were plotted to analyze 1-, 2-, and 3-year OS and compare consistency between nomogram-predicted and actual survival. Decision curve analysis (DCA) was performed to confirm the clinical usefulness of our model.

### Statistical analyses

All data were described as categorical values expressed as frequencies with percentage. Comparisons of baseline characteristics between training and external validation cohorts were performed using Mann–Whitney *U* or chi-squared tests as appropriate.

Survival curves were plotted using the Kaplan–Meier method, stratified according to clinical variables and compared with the log-rank test. Univariate and multivariate Cox regression analyses were employed to identify risk factors independently associated with OS. Variables with *P*-values ≤0.100 on univariate Cox regression analysis were then analyzed using multivariate Cox regression analysis; hazard ratios (HR) were calculated with two-sided 95% confidence intervals (CI).

Statistical significance for all variables was considered at *P *< 0.050 (two-sided). All statistical analyses were performed using either SPSS 22.0 (IBM Corporation, Chicago, United States) or R 3.6.1 (The R Project for Statistical Computing, Texas, United States; http://www.r-project.org) software.

## Results

### Patient characteristics

Available data on 56,376 LSCC patients from January 2010 to December 2015 were collected from the SEER database. After considering aforementioned inclusion and exclusion criteria, our final cohort consisted of 8,078 LSCC patients. Patients enrolled in this study were randomized into training (*n *= 5,656) or validation (*n *= 2,422) cohorts. Details concerning patient inclusion and exclusion are shown in Supplementary Table S1. Patient demographic and clinicopathological characteristics are summarized in Table 1. Among all 8,078 patients included, 4,840 (59.9%) were male and 3,238 (40.1%) were female. The number of patients aged less than 67 years, between 67 and 77 years and over 77 years were 2,894 (35.8%), 3,820 (47.3%) and 1,364 (16.9%), respectively. Among all enrolled patients, more were diagnosed with upper lobe malignancy (58.8%), at T1 stage (50.3%), at N0 stage (83.4%) and underwent pulmonary lobectomy (70.9%). In addition, age composition significantly differed among training and validation cohorts (*P *= 0.010).

**Table 1:** Characteristics of LSCC in training and validation cohorts.

Characteristic	Training cohort (*n *= 5,656)		Validation cohort (*n *= 2,422)		*P*-value
	*N*	%	*N*	%	
**Age at diagnosis (year)**					0.010
<67	2,074	36.7	820	33.9	
66–77	2,666	47.1	1,154	47.6	
>77	916	16.2	448	18.5	
**Sex**					0.184
Male	3,362	59.4	1,478	61.0	
Female	2,294	40.6	944	39.0	
**Race**					0.339
White	4,953	87.6	2,149	88.7	
Black	444	7.9	171	7.1	
Other	259	4.6	102	4.2	
**Marital status at diagnosis**					0.563
Married	3,209	56.7	1,391	57.4	
Unmarried	2,447	43.3	1,031	42.6	
**Insurance status at diagnosis**					0.570
Insured	5,572	98.5	2,390	98.7	
Uninsured	84	1.5	32	1.3	
**Laterality**					0.848
Right	3,140	55.5	1,339	55.3	
Left	2,516	44.5	1,083	44.7	
**Tumor primary site**					0.443
Main bronchus	67	1.2	38	1.6	
Upper lobe	3,347	59.2	1,406	58.1	
Middle lobe	1,858	32.9	823	34.0	
Lower lobe	232	4.1	89	3.7	
Other	152	2.7	66	2.7	
**Differentiation**					0.370
I (well-differentiated)	153	2.7	81	3.3	
II (moderately differentiated)	2,784	49.2	1,162	48.0	
III (poorly differentiated)	2,674	47.3	1,161	47.9	
IV (undifferentiated)	45	0.8	18	0.7	
**T stage**					0.135
T1	2,885	51.0	1,182	48.8	
T2	1,637	28.9	710	29.3	
T3	761	13.5	369	15.2	
T4	373	6.6	161	6.6	
**N stage**					0.249
N0	4,698	83.1	2,037	84.1	
N1	958	16.9	385	15.9	
**Surgery type**					0.550
Lobectomy	4,002	70.8	1,727	71.3	
Pneumonectomy	724	12.8	289	11.9	
Sublobectomy	930	16.4	406	16.8	
**Radiotherapy type**					0.625
No/Unknown	5,074	89.7	2,164	89.3	
Yes	582	10.3	258	10.7	
**Chemotherapy**					0.501
No/Unknown	4,342	76.8	1,876	77.5	
Yes	1,314	23.2	546	22.5	

LSCC, lung squamous cell carcinoma.

### Nomogram variable screening

Univariate Cox regression analysis for OS revealed that age (>77: HR 1.74; 95% CI: 1.54–1.95; *P *< 0.001), sex (male: HR 1.27; 95% CI: 1.16–1.39; *P *< 0.001), marital status (unmarried: HR 1.12; 95% CI: 1.03–1.22; *P *= 0.008), T stage (T2: HR 1.27; 95% CI: 1.15–1.40; *P *< 0.001), N stage (N1: HR 1.42; 95% CI: 1.28–1.57; *P *< 0.001), surgery type (pneumonectomy: HR 1.52; 95% CI: 1.35–1.70; *P *< 0.001) and radiotherapy (HR 1.61; 95% CI: 1.43–1.81; *P *< 0.001) were significant influencing factors. Multivariate Cox regression analysis confirmed that age (>77: HR 1.90; 95% CI: 1.68–2.15; *P *< 0.001), sex (male: HR 1.30; 95% CI: 1.19–1.43; *P *< 0.001), marital status (unmarried: HR 1.20; 95% CI: 1.10–1.31; *P *< 0.001), T stage (T2: HR 1.25; 95% CI: 1.13–1.39; *P *< 0.001), N stage (N1: HR 1.27; 95% CI: 1.14–1.42; *P *< 0.001), surgery type (pneumonectomy: HR 1.24; 95% CI: 1.09–1.40; *P *= 0.001) and radiotherapy (HR 1.37; 95% CI: 1.21–1.55; *P *< 0.001) were significant predictors of OS (Table 2).

**Table 2:** Univariate and multivariate Cox regression analyses of OS predictors in LSCC patients.

Characteristics	Univariable analysis		Multivariable analysis	
	HR (95% CI)	*P* value	HR (95% CI)	*P* value
**Age at diagnosis (years)**				
<67	1.00	-	1.00	-
66–77	1.27 (1.15, 1.4)	<0.001	1.41 (1.28, 1.56)	<0.001
>77	1.74 (1.54, 1.95)	<0.001	1.90 (1.68, 2.15)	<0.001
**Sex**				
Female	1.00	-	1.00	-
Male	1.27 (1.16, 1.39)	<0.001	1.30 (1.19, 1.43)	<0.001
**Race**				
Black	1.00	-	-	-
White	1.07 (0.91, 1.25)	0.436	-	-
Other	0.95 (0.74, 1.23)	0.704	-	-
**Marital status at diagnosis**				
Married	1.00	-	1.00	-
Unmarried	1.12 (1.03, 1.22)	0.008	1.20 (1.10, 1.31)	<0.001
**Insurance status at diagnosis**				
Insured	1.00	-	-	-
Uninsured	0.97 (0.68, 1.39)	0.879	-	-
**Laterality**				
Left	1.00	-	-	-
Right	1.02 (0.93, 1.11)	0.709	-	-
**Primary tumor site**				
Main bronchus	1.00	-	-	-
Upper lobe	0.79 (0.54, 1.14)	0.206	-	-
Middle lobe	0.89 (0.61, 1.30)	0.550	-	-
Lower lobe	0.69 (0.45, 1.06)	0.092	-	-
Other	1.14 (0.74, 1.76)	0.559	-	-
**Differentiation grade**				
II (moderately differentiated)	1.00	-	-	-
I (well-differentiated)	0.99 (0.76, 1.30)	0.958	-	-
III (poorly differentiated)	1.08 (0.99, 1.17)	0.093	-	-
IV (undifferentiated)	1.35 (0.88, 2.09)	0.168	-	-
**T stage**				
T1	1.00	-	1.00	-
T2	1.27 (1.15, 1.40)	<0.001	1.25 (1.13, 1.39)	<0.001
T3	1.54 (1.36, 1.74)	<0.001	1.49 (1.31, 1.70)	<0.001
T4	2.42 (2.09, 2.80)	<0.001	2.22 (1.90, 2.61)	<0.001
**N stage**				
N0	1.00	-	1.00	-
N1	1.42 (1.28, 1.57)	<0.001	1.27 (1.14, 1.42)	<0.001
**Surgery type**				
Lobectomy	1.00	-	1.00	-
Pneumonectomy	1.52 (1.35, 1.70)	<0.001	1.24 (1.09, 1.40)	0.001
Sublobectomy	1.29 (1.16, 1.44)	<0.001	1.38 (1.23, 1.55)	<0.001
**Radiotherapy**				
No	1.00	-	1.00	-
Yes	1.61 (1.43, 1.81)	<0.001	1.37 (1.21, 1.55)	<0.001
**Chemotherapy**				
No	1.00	-	-	-
Yes	1.03 (0.93, 1.14)	0.555	-	-

Data were presented as hazard ratios (HR) and 95% confidence intervals (CI). OS, overall survival; LSCC, lung squamous cell carcinoma.

### Nomogram construction and validation

**Figure 1:**
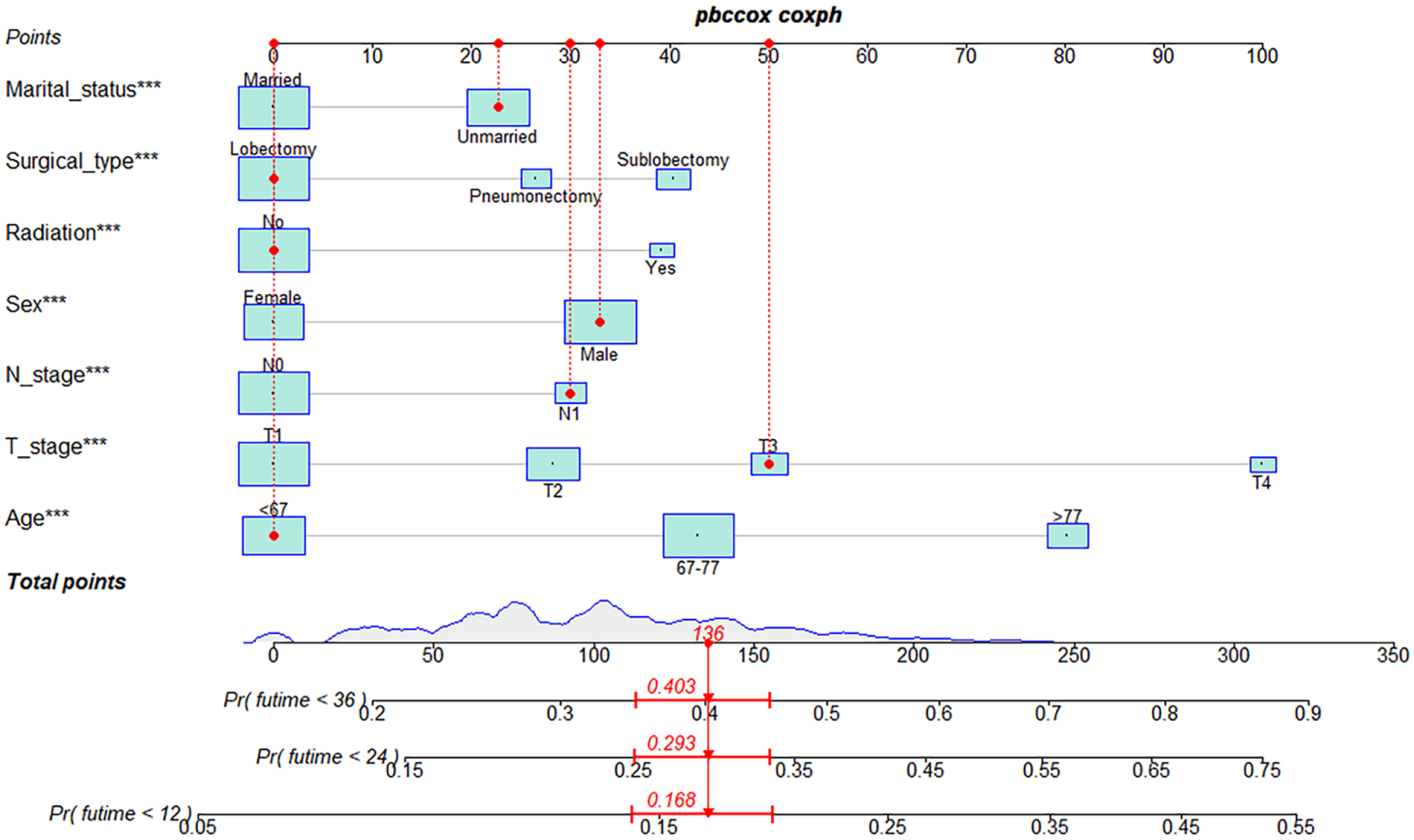
Nomograms to predict 1-, 2- and 3-year OS for postoperative LSCC patients. OS, overall survival; LSCC, lung squamous cell carcinoma.

Seven independent risk factors (age, sex, marital status, T stage, N stage, surgical type, and radiotherapy) were considered as prognostic predictors for nomogram construction. T stage had the greatest beta, while marital status had the least. Figure 1 details one LSCC patient who possessed the following characteristics: male; aged <67 years; unmarried; T3N1M0; prior lobectomy; no prior radiotherapy. In this example, the patient scored 136 points and thus was predicted to have probabilities of 1-, 2- and 3-year OS of 0.832, 0.707 and 0.597, respectively.

**Figure 2:**
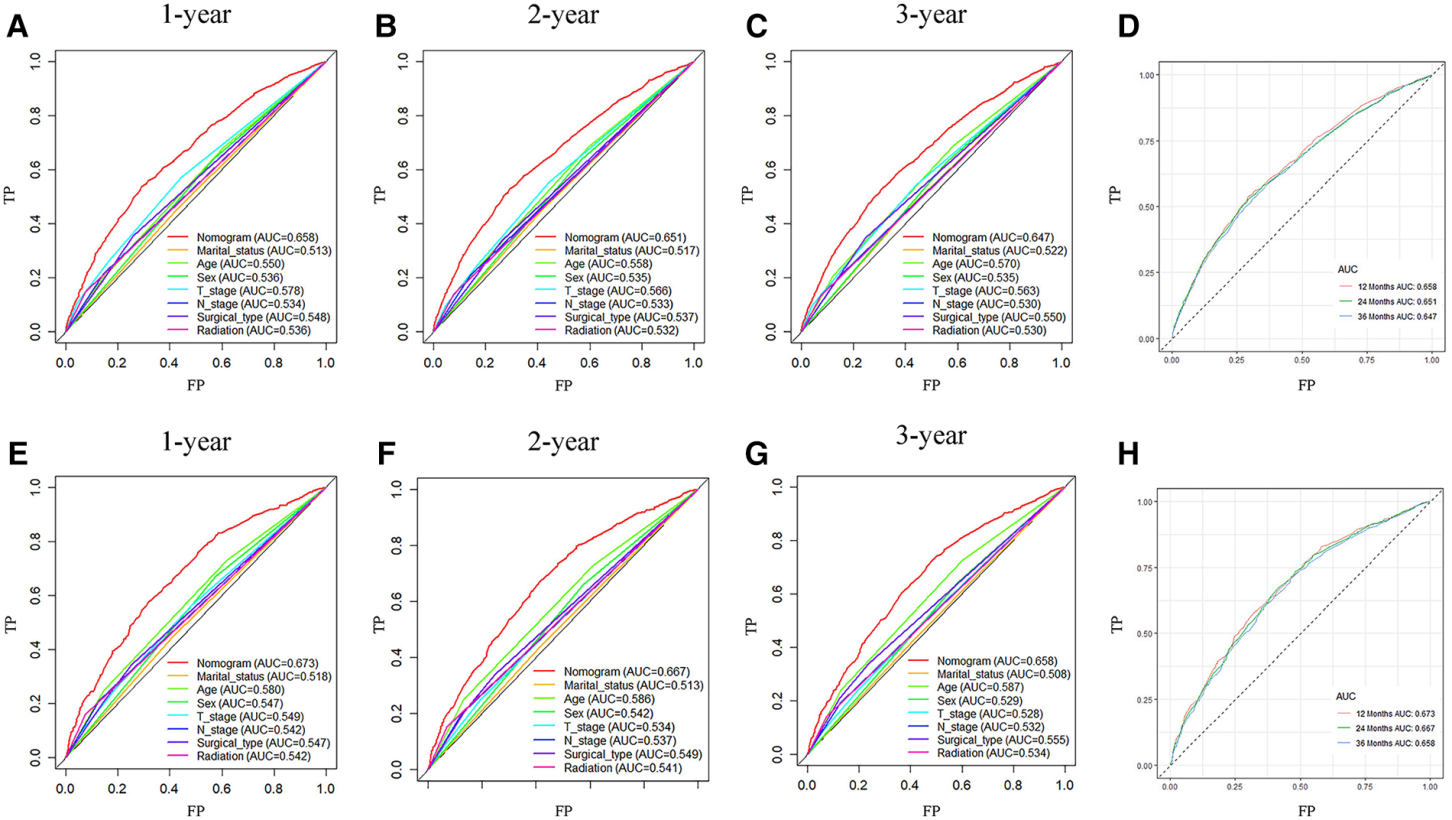
ROC curves of nomogram prediction of prognoses in training and validation cohort patients. (**A–D**) ROC curve for 1-, 2- and 3-year timepoints in the training cohort. (**E–H**) ROC curve for 1-, 2- and 3-year timepoints in the validation cohort. ROC, receiver operating characteristic curve; AUC, area under the ROC curve; TP, true positive rate; FP, false positive rate.

**Figure 3:**
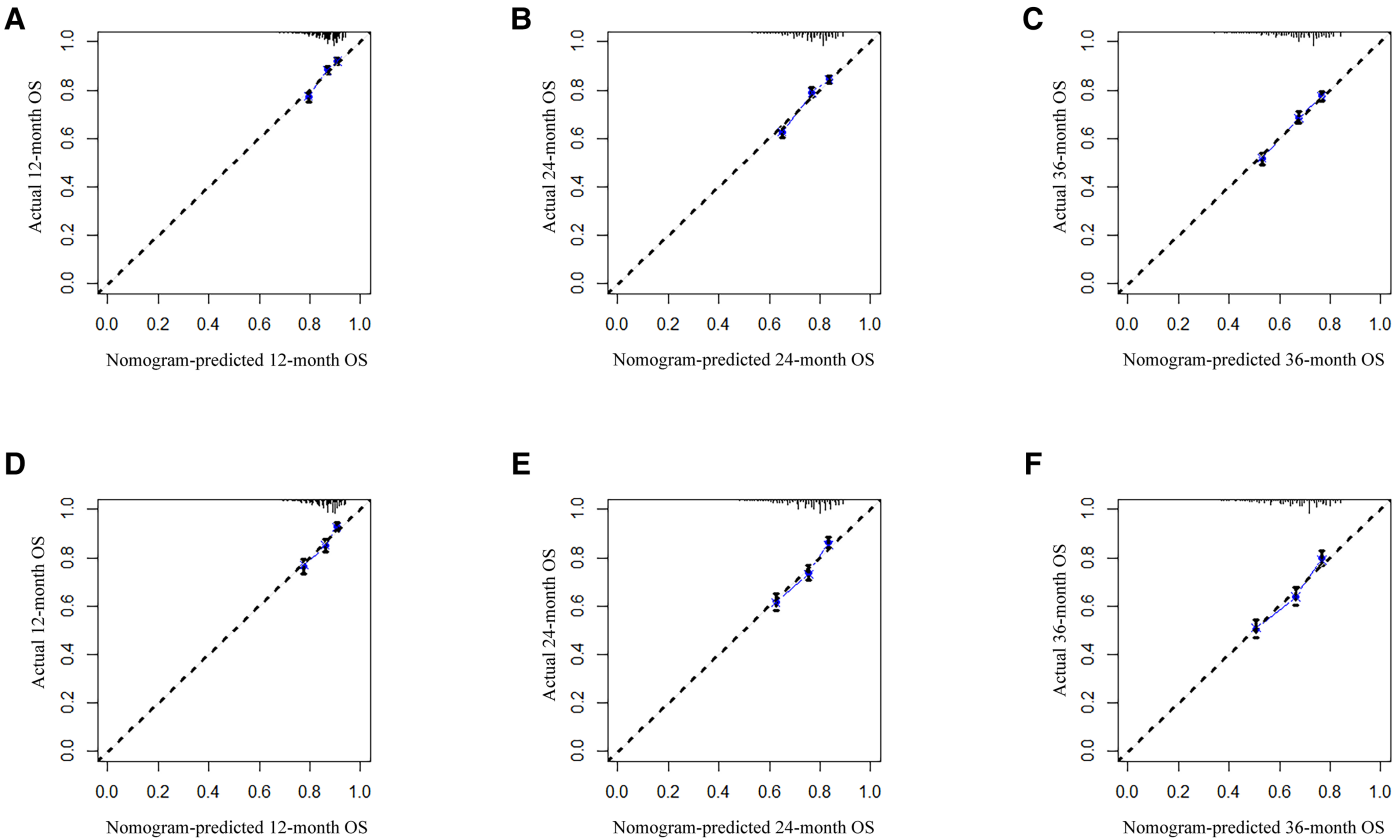
Calibration curves for predicting patient OS at 1-, 2- and 3-year timepoints in training (**A–C**) and validation (**D–F**) cohorts, respectively. The 45-degree line represents an ideal match between actual (*y*-axis) and predicted (*x*-axis) survival. OS, overall survival.

**Figure 4:**
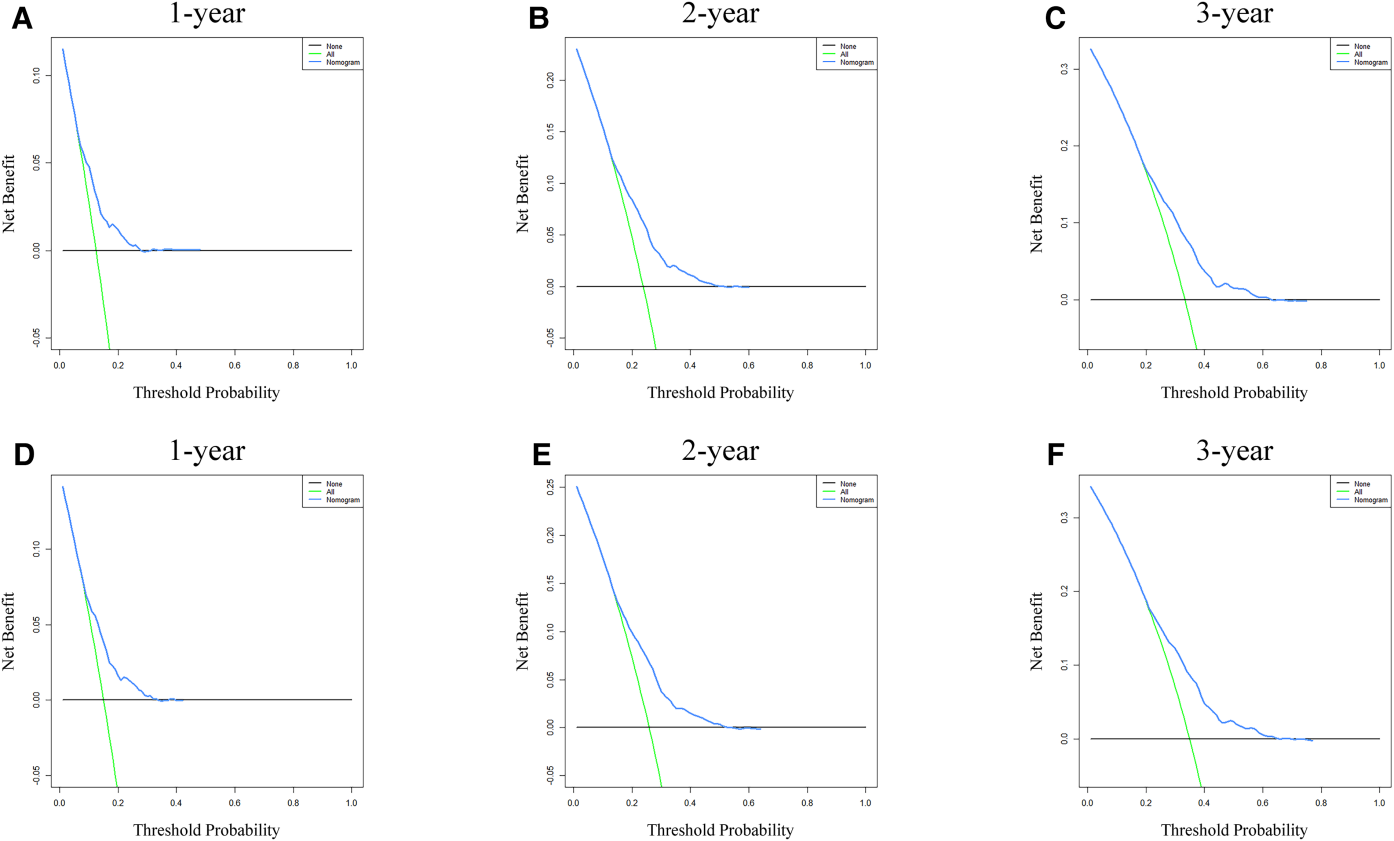
Nomogram DCA for survival prediction of LSCC patients. (**A–C**) 1-, 2- and 3-year survival benefit in training cohort patients. (**D–F**) 1-, 2- and 3-year survival benefit in validation cohort patients. DCA, decision curve analysis; LSCC, lung squamous cell carcinoma.

Analysis of AUC values revealed OS probabilities of 0.658, 0.651 and 0.647 for the training cohort and 0.673, 0.667 and 0.658 for the validation cohort at 1-, 2- and 3-year timepoints, respectively (Figure 2). The c-index of the training set was 0.624 (95% CI: 0.599–0.649), and the c-index of the validation set was 0.640 (95% CI: 0.605–0.675). Moreover, calibration curve plotting revealed good consistency between nomogram-predicted and observed survival probabilities at 1-, 2- and 3-year timepoints for both cohorts (Figure 3). Importantly, DCA confirmed our nomogram to be clinically useful (Figure 4).

### Risk stratification and survival analysis

**Figure 5:**
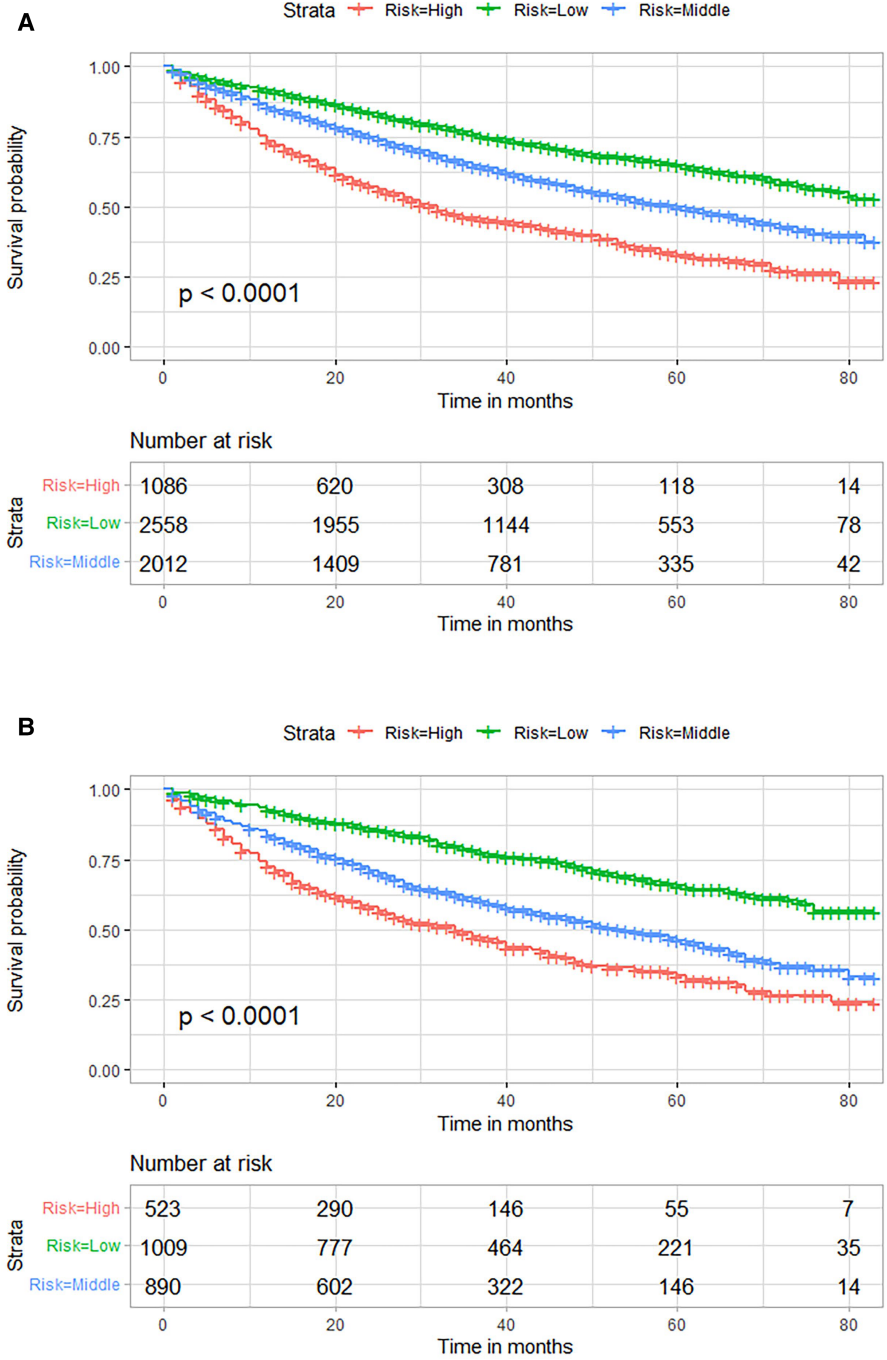
Kaplan–Meier OS curves for risk stratification based on total nomogram scores in the training (**A**) and external validation (**B**) cohorts. OS, overall survival.

Risk stratification based on total score assigned using our established nomogram was performed to distinguish prognostic risk profiles (Figure 5). Patients were divided into low (total points <95), middle (total points 95≤ and <143) or high (total points ≥143) risk groups. Kaplan-Meier survival curves revealed significant difference in OS among these risk groups (*P *< 0.001). Low-risk group patients had higher OS probabilities as compared to middle- and high-risk group patients.

## Discussion

In this study, a nomogram was constructed and validated to predict 1-, 2- and 3-year OS for postoperative LSCC patients. Patient age, sex, marital status, T stage, N stage, surgery type and use of radiotherapy were identified as clinical risk factors on variable screening. Our nomogram showed favorable discrimination and calibration values. Currently, for N2 patients with single-station metastasis, surgery and (neo)adjuvant chemotherapy can be considered. However, for N2 patients with multi-station metastasis, surgery is not recommended (13). In addition, the SEER database only provides information on N2 without distinguishing between single-station or multi-station metastasis, so we did not include N2 patients in our study. Before establishing a nomogram model, blindly including cases with unclear variables will affect the accuracy of the model. Therefore, it is necessary to clarify the patient's information such as marital and insurance status.

Survival prediction tools such as nomograms facilitate estimation of a probability that a clinical event, such as cancer recurrence or death, can occur in a patient (14). Nomograms, which are graphical tools based on regression equations, are becoming increasingly popular for presenting the results of prediction models due to their convenience and visual appeal (15). While efforts to develop predictive nomograms for lung cancer have intensified over recent years, relevant research to date studied NSCLC and LUAD due to NSCLC accounting for approximately 85% of lung cancers and LUAD being the most abundant type of NSCLC (16). Studies investigating LSCC prognosis, however, remain scarce. Zhang et al. (17) constructed a nomogram to accurately predict the incidence of brain metastasis in LSCC patients and facilitate early identification of high-risk individuals. Our study, however, excluded M1, N2 and N3 stage LSCC patients not eligible for surgical treatment and focused on evaluating patient survival. Zheng et al. (18) performed a nomogram study similar to ours which satisfactorily predicted 3-, 5- and 7-year cancer-specific OS rates for LSCC patients. That study employed limited methods to evaluate the quality of predicted models and evaluated all stage lung cancer patients regardless of their eligibility for surgery. Importantly, LSCC and LUAD significantly differ in terms of genetics and molecular characteristics, such as epidermal growth factor receptor gene mutations, as well as prognosis (19). With the advent of low-dose computed tomography, identification of early-stage lung cancer has become more common and thus improved chances of successful conservative surgical management as well as better long-term survival (20). As cancer patient prognosis is highly associated with tumor stage and varies greatly, separate evaluation of operable and inoperable LSCC cases is warranted (21). Here, we predicted 1-, 2- and 3-year LSCC patient OS based on analysis of data obtained from the SEER database.

Li et al. (22) previously reported 8 RNA binding proteins to serve as prognosis-related hub genes, which were used to construct a nomogram for predicting LUAD patient OS. Liu et al. (23) identified 33 autophagy-associated genes that could dichotomize patients with significantly different OS and independently predict OS in LUAD and LSCC patients, respectively. Most of those nomogram-related prognostic studies utilized ROC curve analysis, calibration curve plotting and DCA to assess predictive performance. Prior survival-related nomograms were also based on transcription- or translation–level data. However, accurate data sequencing can be difficult as sequencing platforms may be not accessible to many medical institutions and patients. In contrast, our predictive nomogram was constructed based on readily available patient clinicopathological and demographic characteristics.

Multivariable Cox regression analysis revealed that patient age, sex, marital status, T stage, N stage, surgery type and radiation treatment were significantly associated with OS. Patients aged >77 years had a significantly higher risk of death as compared to those aged <67, implying that advanced age is a poor prognostic factor LSCC patients, consistent with prior studies (24). Many studies reported sex differences in survival, with female lung cancer patients have higher OS rates (25). Our nomogram confirmed that male sex significantly negatively correlated with survival probability. Marital status was also reported to significantly associate with survival in cancer patients (26). The survival advantage linked to marriage is frequently explained by greater social support, improved mental well-being, and practical assistance, including guidance in navigating the healthcare system (27, 28). Similarly, TNM stage, a well-known prognostic factor for malignancy, was reported to significantly influence prognosis for NSCLC patients (29). Resectable (stage I, II and occasionally III) cases of lung cancer are generally most effectively treated by surgical removal of the tumor *via* pneumonectomy, lobectomy or sublobectomy (30). Our findings revealed that lobectomy scored highest among surgical treatment methods, followed by pneumonectomy and sublobectomy; nomogram scores negatively correlated with prognosis. Lung resection significantly impacts both pulmonary function as well as OS; different surgical approaches are known to differently influence long-term survival (31). Although lobectomy was reported to associate with lower rates of surgical complications and operative mortality as compared to pneumonectomy, such outcomes were likely noted due to smaller surgical resection volumes and more preservation of normal lung tissue (31). Types of surgical treatments were previously compared in the context of long-term LSCC patient survival. A retrospective study by Gezer et al. (32) reported that LSCC patients treated with sleeve lobectomy had lower mortality, better lung function and higher quality of life as compared to patients treated with standard pneumonectomy.

Importantly, earlier and more accurate prediction of long-term survival in preoperative LSCC patients would provide more time for physicians to offer individualized treatment strategies. Our study possessed the advantage of a large sample size that included 8,078 LSCC patients, thus allowing robust prognostic model evaluation. As this study was population-based, our findings are representative of real-world clinical practice. Moreover, compared to nomograms based on genomic data, our model can be more readily adapted for use in a variety of clinical settings that vary from rural clinics to major tertiary hospitals.

This study was not, however, without limitations. First, although the SEER database pools patients from 18 registries, some data may not be relevant to individuals who are members of populations not included in SEER data. Prospective multicenter studies are warranted to confirm our findings and future studies can focus on external validation using data from other sources or patients in our ountry and hospital. The original data in the database categorized “none” and “unknown” into one group, making it impossible to distinguish between them. Second, nomogram variables derived from SEER data did not consider clinical parameters such as symptoms, signs, comorbidities, prior cigarette use, recent weight loss, family history of cancer, radiotherapy type and occupational exposures. In addition, clinical data available to us were not universally reasonable: for example, SEER data tumor staging was performed based on 6th and 7th edition TNM classification guidelines set forth by the AJCC, which differ from those of the 8th edition. Although we attempted to use recent staging guidelines, clinical accuracy was likely nevertheless affected. Factors such as classification of patients treated and not treated with chemotherapy into the same category similarly suggest a possibility of result bias. Finally, the assumption that various predictors interact in additive and linear manners resulted use of traditional Cox regression analysis in the development of our predictive model. As such, the predictive power of the nomogram could be limited due to inherently non-linear and multifactorial interactions among predictors (33).

## Conclusion

Here, we identified patient age, sex, marital status, T stage, N stage, surgery type and radiotherapy to have been independent predictive factors of prognosis for postoperative SEER database LSCC patients. We constructed and validated a nomogram for predicting OS in this patient population and confirmed its clinical usefulness in assisting evaluation of prognosis and tailoring individualized therapeutic strategies to improve patient outcomes.

## Data Availability

The original contributions presented in the study are included in the article/**Supplementary Material**, further inquiries can be directed to the corresponding author.
